# Some Points to Be Borne in Mind

**Published:** 1919-10-11

**Authors:** 


### PHYSIOLOGY AND MEDICINE.

### Some Points to be Borne in Mind.

The medical schools of the country are at the moment launched upon what is, or is hoped to be, a new phase of medical teaching, and although the heads of affairs undoubtedly have a perfectly clear conception of the exact direction in which the changes must lie, it will be by no means easy to communicate the true essentials to the student himself, who in a great number of instances comes into his new profession replete with ideas and ambitions built up upon hearsay of medical traditions of the past. In a short note it is impossible to deal with more than one small corner of so extensive a subject, but in as much as the student himself must understand the true meaning of any such developments before he can drop readily into the successful paths, it is well worth while to draw his attention to some few of the principles which are necessarily concerned in the new phases around him.

### Physiology in the Past.

Regarding the subject of our present choice, one cannot do better than refer back to the words of Sir Edward Sharpey Schafer,* as reprinted from the *Edinburgh Medical Journal*. Nowadays one does not so frequently hear the complaint that too much physiology is taught to medical students. The danger lurking in the older view is becoming daily more obvious, and even the laity are awakening to the fact that, deprived of physiology, medicine can make no progress whatever. Already one of the leading London papers has published an article in which the dependency upon it of the whole superstructure of medicine and surgery was definitely insisted upon. The great majority of practising medical men—and it is they who so markedly influence the earliest student outlook—are, however, seldom heard to express this opinion, their own scanty physiological training was usually promptly forgotten, and they never truly realised that every disease if its conditions are to be understood must be the subject of physiological study. Relatively few of the modern clinicians have ever had the opportunity of carrying out, along the lines of this science, any real investigational work necessarily implying long hours of laboratory work. The author aptly summarises the situation in asking: "How should the clinician know, and why should he care, whether his methods are scientific or not, if they prove sufficiently successful to enable him to gain a reputation as a practitioner and a more or less lucrative income? He may well believe that if he himself has been able to acquire skill and experience in the diagnosis and treatment of disease by methods traditionally handed down from preceding generations, this is going to be the procedure until the end of time, and that these methods would succeed in turning others into just as good practitioners as himself. And very probably they would!"

* Presidential address delivered to the Edinburgh University Physiological Society, January 16, 1919.

It is impossible justly to include the whole profession in such indictment, but nevertheless there is no doubt that before a great many men whose real aim is to progress, and particularly before the new generation of students, we should most emphatically bring Sir Edward Schafer's proposition that physiology is the pivotal subject around which all the medical sciences are centred, and which furnishes a basis upon which the whole of medicine and surgery is founded. This statement admitting of such convincing proof as the author provides, is incidentally impossible reasonably to disprove from any aspect whatsoever. The science of the living organism is so obviously essential to either the physician or surgeon, and yet we have in the past given enormously more time to the study of the dead—namely, anatomy. Even then it is most unfortunate that the study of anatomy is confined so exclusively to the dead subject. Perhaps it may help the student to realise that the time-honoured dissecting-room methods fall far short of the ideal when he notes later that so little which is really useful to surgery and medicine is learned by the older methods of teaching anatomy in spite of the great time taken—all at the expense of physiology, too—that it becomes almost invariably necessary to have special courses in so-called medical and surgical anatomy in order to supplement the deficiency. Yet we read that out of 2,100 working hours of the first two years of the curriculum each student has been expected to give 1,300 to anatomy and only 260 to physiology.

### Macroscopic and Microscopic.

Another branch must also be considered in its anatomical-physiological relationship — namely, histology. "As to this whatever has been stated regarding the position of macroscopic anatomy applies equally, perhaps more so, to microscopic anatomy. Its chief interest lies in its utility for the elucidation of physiological problems. It has therefore been a sound tradition in Great Britain to place the teaching of microscopic anatomy with the physiologist, rather than, as has been done in Germany, with the anatomist. There has been lately a tendency on the part of certain British physiologists to neglect or belittle this important asset to their methods of inquiry, but the best physiologists have usually been good histologists, and it is an indisputable fact that many of the most important advances in histology have been made by physiologists in pursuit of purely physiological problems."

But to return to physiology itself, it is a vast subject with so many ramifications which are perhaps not fully realised until the later years of medical training, that the student had best remember that if he could actually devote an academic year to its study he might then have a chance to gain a knowledge of true practical utility in application to his later studies of disease. Actually he will fall far short of this time, and all the more necessary does it

**Physiology and Medicine**—(*continued*).

thus become that he should not treat it lightly as a subject to be touched upon and then forgotten. Everything a student learns before he comes to physiology leads up to it, and owes its main value to that circumstance. "On the other hand, physiology through its sister sciences, pathology and pharmacology—between which and physiology there is no dividing line—leads to clinical medicine and surgery. And besides this connection through pathology and pharmacology, physiology has a still more intimate relation to medical and surgical practice, for without a present and accurate knowledge of the normal functions of the body, the investigation of abnormalities is impossible. And this is just as true for surgery as for medicine; partly because most surgical cases are, in the first place, medical cases, partly because the surgeon, as well as the physician, is constantly coming across problems which are purely physiological in character."

IN CONCLUSION.

But everything one can say with regard to relationship to medicine and surgery as general subjects applies even more forcibly to the value of physiological understanding as brought to bear upon the professional branches in which men are inclined ultimately to specialise. The author mentions as examples midwifery, diseases of the nervous system, affections of the eyes, diseases of the secreting glands, and, of more recent application, the derangements of the endocrine organs. In reality there is no corner so remote in clinical work that the illuminating aid of physiology cannot reach it, although time must yet pass before its mission is complete. The teaching of applied physiology —that is, actual medicine from a physiological point of view—must be in the hands of clinical teachers who are themselves trained physiologists. Probably they should largely owe their selection to this training rather than to their success as brilliant operators or fashionable physicians. Happily reforms and reconstruction are occurring everywhere, and the student of to-day will find many of the authors' wishes fulfilled, and for success has probably but to act largely in accordance with such aims.

